# Iatrogenic Bladder Injury During Laparoscopic Hysterectomy: A Case Report and Discussion of Anatomic Variations

**DOI:** 10.7759/cureus.56556

**Published:** 2024-03-20

**Authors:** Srihita Patibandla, Syed Mohammed Amanuddin, Ali Z Ansari, Ali Saeed, Kurt Kratz

**Affiliations:** 1 Obstetrics and Gynecology, William Carey University College of Osteopathic Medicine, Hattiesburg, USA; 2 Pathology, Merit Health Wesley, Hattiesburg, USA

**Keywords:** laparoscopically assisted vaginal hysterectomy, vaginal hysterectomy, iatrogenic perforation, intraoperative complications, multiple uterine fibroids, abnormal uterine bleeding, total abdominal hysterectomy, total laparoscopic hysterectomy, anatomical variations, iatrogenic bladder injury

## Abstract

Hysterectomy, one of the most common surgical procedures performed in women worldwide, assumes a very important role in the definitive management of diverse gynecologic conditions. This case report presents a compelling instance of an iatrogenic bladder perforation that occurred during laparoscopically assisted vaginal hysterectomy in a 47-year-old woman with a high body mass index, extensive surgical history, and postural orthostatic tachycardia syndrome. Despite considerable preoperative planning and the use of minimally invasive techniques, the occurrence of physician-induced bladder perforation highlights the significance of understanding anatomical relationships and variations. The patient’s previous abdominal surgeries including two cesarean sections, appendectomy, and cholecystectomy likely contributed to scar formation and adhesions, making dissection challenging. The case report and following discussion delve into anatomical variations, as well as the diagnosis and management of iatrogenic bladder injuries. The presented case serves as a valuable addition to the literature, contributing insights into the challenges and considerations surrounding urinary tract injuries during hysterectomy. This paper aims to review current research and guide practicing obstetricians and gynecologists in the management of intraoperative bladder injuries.

## Introduction

Hysterectomies are one of the most common surgeries performed within the specialty of obstetrics and gynecology, with approximately one in every nine women in the United States undergoing the procedure [1]. Some of the most common indications for hysterectomy are leiomyomata, adenomyosis, endometrial hyperplasia, uterine prolapse, dysmenorrhea, cervical intraepithelial neoplasia, and dysfunctional uterine bleeding [2]. Some of the most common complications encountered with hysterectomies are post-operative fever and infection (4-25%), ureteral injury (0-1.7%), bladder injury (0.5-2%), bowel injury (0.1-1%), hemorrhage (1-3%), thromboembolic disease (0.2-0.4%), pelvic organ prolapses (3.2-17.2%), impaired sexual function, early menopause, and psychological effects [3-5]. Having a thorough understanding of the anatomy of the vagina, rectum, bladder, bowels, and surrounding structures, along with an awareness of potential anatomic variations can prevent potential iatrogenic complications during surgery [4,6]. Thorough pre-operative planning using imaging and staying updated on indications for surgery and alternative treatment options can also be of significant importance [6].

This paper presents the case of a bladder perforation that occurred during laparoscopically assisted vaginal hysterectomy and discusses literature regarding anatomical considerations and management approaches. Given the rarity of the incidence of bladder injuries during hysterectomies, this paper aims to provide an understanding of their management from gynecological and urological perspectives, which is important for physicians who are faced with these conditions during surgery. In addition, we will delve into intraoperative steps that can be taken to detect urinary tract injuries, particularly in challenging cases where the injury may not be directly visible. By reviewing these intraoperative measures, this paper aims to provide valuable insights into improving the detection and management of urinary tract injuries during hysterectomy.

## Case presentation

**Figure 1:**
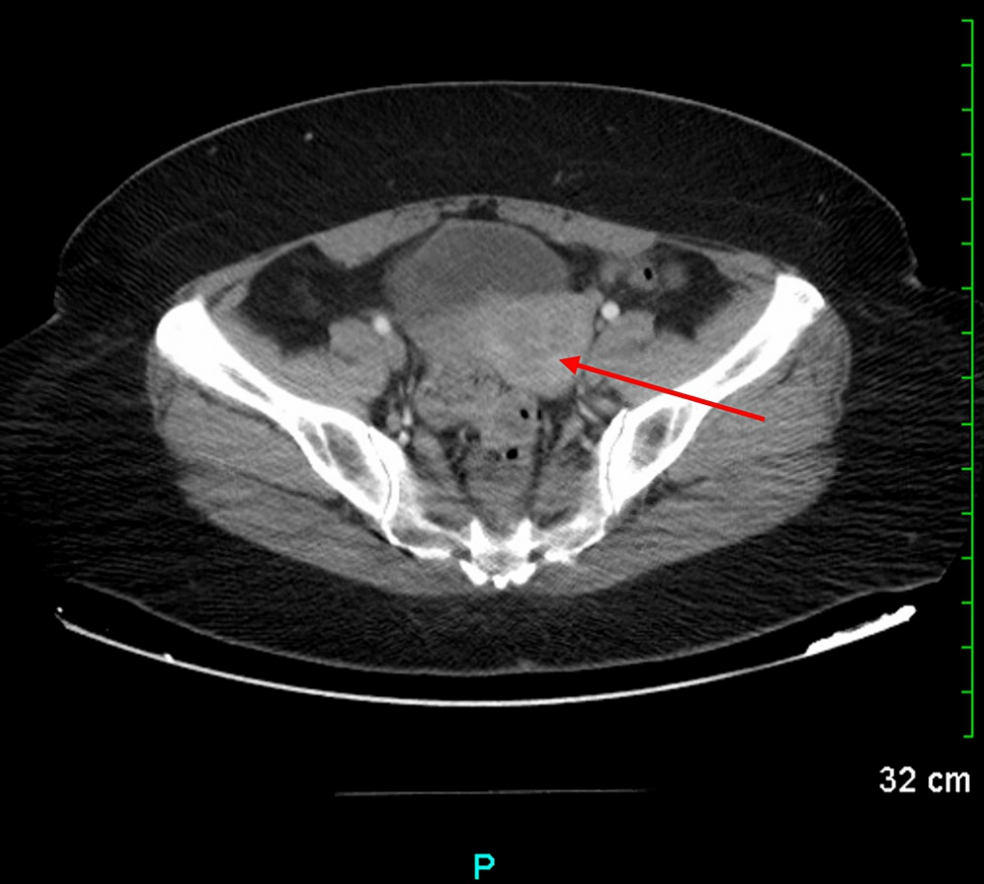
CT scan of the abdomen and pelvis with contrast taken prior to the patient’s endometrial ablation procedure The red arrow shows a lobular contour to the uterus suggestive of leiomyomas.

The patient is a 47-year-old white gravida 3, para 2, abortus 1 female, with a body mass index (BMI) of 41.2 kg/m^2^ and a past medical history of postural orthostatic tachycardia syndrome (POTS), fibromyalgia, and lupus. Her past pregnancies included two full-term cesarean section deliveries, one at 40 weeks and one at 37 weeks gestation. Her other surgical history included a cholecystectomy, appendectomy, spinal surgery, and ankle surgery. The patient had a history of medical management-resistant menorrhagia, with regular monthly cycles with heavy bleeding and dysmenorrhea lasting an average of 10 days at a time. She was not on any form of contraception and has not been recently sexually active. The patient had a thin endometrial thickness that was not consistent with endometrial cancer. Ultrasound imaging revealed a normal uterine contour, while computed tomography (CT) showed a lobular contour to the uterus suggestive of possible leiomyomas, consistent with her CT from a year ago (Figure 1). She failed both a trial of Provera (medroxyprogesterone acetate) and Megace (megestrol acetate) and presented to the hospital for a laparoscopically assisted vaginal hysterectomy, after not having success with an endometrial ablation as well the previous year. With no desire for future fertility, the patient requested definitive surgery for her condition. Prior to the procedure, an indwelling urinary catheter was placed.

In the operating room, the patient underwent general anesthesia. During the procedure, a moderate amount of scar tissue was noted, particularly on the left side wall, likely secondary to the patient’s previous abdominal surgeries. The scar tissue extended from the vesicouterine peritoneum to the left side wall and was carefully dissected down to the lower uterine segment. Attention was then turned to the contralateral side. The round ligament was transected, and the anterior sheath of the broad ligament was dissected down to the beginning of the vesicouterine peritoneum. It was initially noted that there was what appeared to be the ureter in the anterior distal portion on the right side. The broad ligament was dissected through, and the tubular structure was then skeletonized up into the lower uterine segment. Here, the structure was identified to be the uterine artery. Continued dissection was performed down through the posterior sheath of the broad ligament. The vesicouterine peritoneum was dissected through the scar tissue down to the Koh cup. Dissection was then performed on the left side down to the uterine artery, which was transected as well. At this point, the ureters were reevaluated and found to be inferior and distal to the planned surgical site. Continued dissection was performed through the uterosacral ligaments. With continuous forward pressure with the Koh cup, the anterior part of the vesicouterine peritoneum and the vesicouterine fascia were brought down. At this time, a small rent was noted in the bladder, which was created during dissection through the scar tissue. Hemostasis was present, and the abdomen was deflated. While routine intraoperative cystoscopy was not performed at our facility, doing so could have aided in the recognition of injury in cases where it is not easily visualized.

Urology was consulted for laparoscopic cystorrhaphy, which confirmed two holes just to the right of the midline of the patient’s bladder within the peritoneum, one 3-4 mm and one 4-5 mm in size, which were subsequently repaired. The 4- to 5-mm hole was repaired using 3-0 Vicryl in a figure-of-eight fashion, overlaid by 2-0 Vicryl in a figure-of-eight fashion. The 3- to 4-mm hole was repaired with 2-0 Vicryl. There was no leakage seen from the bladder after repair. Intraoperative cystoscopy was performed and noted no obvious other injuries, and there was good reflux of urine from both ureteral orifices. An abdominal drain was placed, to be removed if the output is less than 30 cc per shift. The remainder of the hysterectomy was performed without complications, and the patient was transferred to the post-anesthesia care unit (PACU) in stable condition.

The pathology report of the uterus listed gross findings of numerous membranous serosal adhesions near the apex of the corpus and hyperemic endocervix and endometrium, with an endometrial thickness of 0.2 cm. The endometrium had a constricted appearance, and sectioning revealed white, whorled, tan fibroids measuring up to 2 cm occurring in subendometrial locations. Microscopically, the specimen was positive for adenomyosis, leiomyomata, mildly disordered proliferative endometrium, and serosal fibrovascular adhesions, and was negative for cervical dysplasia.

**Figure 2:**
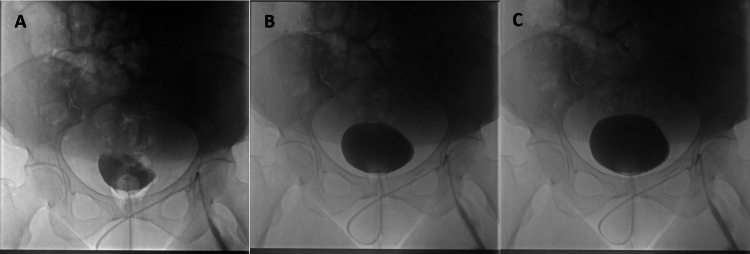
Follow-up cystogram for surgical bladder disruption after 14 days. No mural irregularity or contrast extravasation can be seen. Subfigures A, B, and C show the progressive flow of 250 mL of Cystografin contrast filling the urinary bladder.

In the post-operative period, the patient’s Foley catheter was left in for 14 days per the urologist’s recommendations. She also started a three-day course of Zithromax and Macrobid, which was to be continued until her catheter was removed. She had several episodes of hypotension overnight during the first post-operative night, likely secondary to her POTS. She had no episodes of loss of consciousness. She had moderate pain control and scant vaginal bleeding. She was tolerating a regular diet. Her infraumbilical incision had moderate bleeding on the bandage, but the incision was hemostatic, and her other three incisions were hemostatic as well. Her drain was removed secondary to minimal drainage. Her complete blood count was within normal limits, consistent with her post-operative status, and she had a minimal drop in her hemoglobin levels and a slight increase in white blood cell count as expected. She had adequate urine output of clear yellow, blood-tinged urine. Her home medications were resumed, and midodrine was scheduled to be given as needed for her hypotension secondary to POTS. She was discharged home the following day in stable condition with her catheter. The follow-up cystogram after two weeks was unremarkable (Figure 2).

## Discussion

Anatomical variations

When the decision to perform hysterectomy is made, surgeons must navigate a diverse range of anatomical variations that significantly influence surgical approach and outcomes as misidentification of anatomy can lead to iatrogenic injuries [6]. Variations in uterine size, shape, and position within the pelvis require a tailored approach for each patient. In a literature review by Matsas et al. [6], ureters and uterine arteries were found to be the most vulnerable structures to injury during hysterectomy due to anatomical variations. In particular, it was noted that ureters can present with multiplications, retroiliac positioning, and ureteric diverticula, and uterine arteries can vary in their point of origin [6]. In ureteral multiplication, the ureters are typically contained within a single sheath and the associated blood supply could be compromised. One of the ureters may also be mislabeled as a vessel, cut, and ligated during dissection [7]. Ureteral diverticula or retroiliac ureters may also be confused with vascular structures [7]. The primary vessels of origin of the uterine arteries as concluded in a review by Liapis et al. [8] were the internal iliac artery, the umbilical artery, and the inferior gluteal artery. When encountered with a C-shaped uterine artery variation, Peters et al. [9] describe the key steps in its identification and isolation, approaching the artery origin either from the pararectal space or by utilizing the medial umbilical ligament coursing through the paravesical space. El-Agwany [10] described the case of a patient who underwent hysterectomy for endometrial cancer with an incidental finding of bilateral absent internal iliac arteries with the common iliac artery extending as the external iliac artery. In this variation, the branches of the absent internal iliac artery may arise from the aorta or the external iliac artery, and the uterine artery can be traced from the uterine side, and any branches from the external iliac artery in the pelvis can be normal variations [10]. Pelvic anatomy is not necessarily completely symmetrical on the right and left sides from a laparoscopic view. In some patients, the right ureter runs significantly closer to the infundibulopelvic and uterosacral ligaments than the left ureter, and in others, the right inferior epigastric vessels and umbilical ligament course more laterally than the vessels on the left [11]. Kostov et al. [7] explored in depth other anatomical variations and factors to consider during gynecologic surgery in patients with these variants. Other factors that may make navigating a patient’s anatomy difficult are obesity and adhesions due to difficulty obtaining a clear visual field [12]. These factors likely played a role in the iatrogenic bladder injury found in the presented patient case, given the patient’s BMI of 41.2 kg/m^2^ and extensive surgical history increasing the likelihood of adhesions. The intricate nature of pelvic anatomy poses a significant challenge for surgeons performing hysterectomies. Particular attention and a thorough understanding of anatomy is required for vulnerable structures, especially those that exhibit anatomic variations, to avoid iatrogenic injury.

Diagnosis of iatrogenic bladder perforation

Urinary tract injury is among the most common forms of iatrogenic injury during gynecologic and obstetric operations. When intraoperative injury is suspected, the surgeon must promptly identify the damaged structure and assess the extent of injury. A thorough examination of the entire urinary tract is recommended when injury in one region is identified. Intraoperative bladder injuries should be suspected when there are findings of urine in the operative field, air in the Foley catheter collection bag, or direct visualization of the Foley catheter [13]. In the presented case, the injury was directly visualized during the operation. While the majority of urinary tract injuries can be detected intraoperatively, those that are not recognized typically present in the post-operative period with symptoms such as suprapubic pain, hematuria, oliguria, dysuria, fever, lower back or flank pain, leukocytosis, incontinence, and/or vaginal urinary leak [13]. Labs may show elevated serum creatinine and blood urea nitrogen [13]. A systematic review of various methods for intraoperative evaluation by Siff et al. [14] showed that cystoscopy, oral phenazopyridine and vitamin B, intravenous (IV) methylene blue, IV sodium fluorescein, IV indigo carmine, prophylactic retrograde ureteral stents, and transabdominal Doppler ultrasound were all safe and effective in diagnosing urinary tract injury intra- and post-operatively. A review by Gilmour et al. [15] showed that the use of routine intraoperative cystoscopy during major gynecologic and urogynecologic surgery may prevent sequelae from lower urinary tract injuries. Dumont et al. [16] also found that prophylactic ureteral catheterization is able to significantly enhance the intraoperative diagnosis of iatrogenic ureteral injury and its immediate repair. Consequently, maintaining a low threshold for suspecting urinary tract injury during obstetric and gynecologic surgeries is crucial in prompt identification, timely intervention, and avoiding costly routine screenings.

Management of bladder perforations

**Figure 3:**
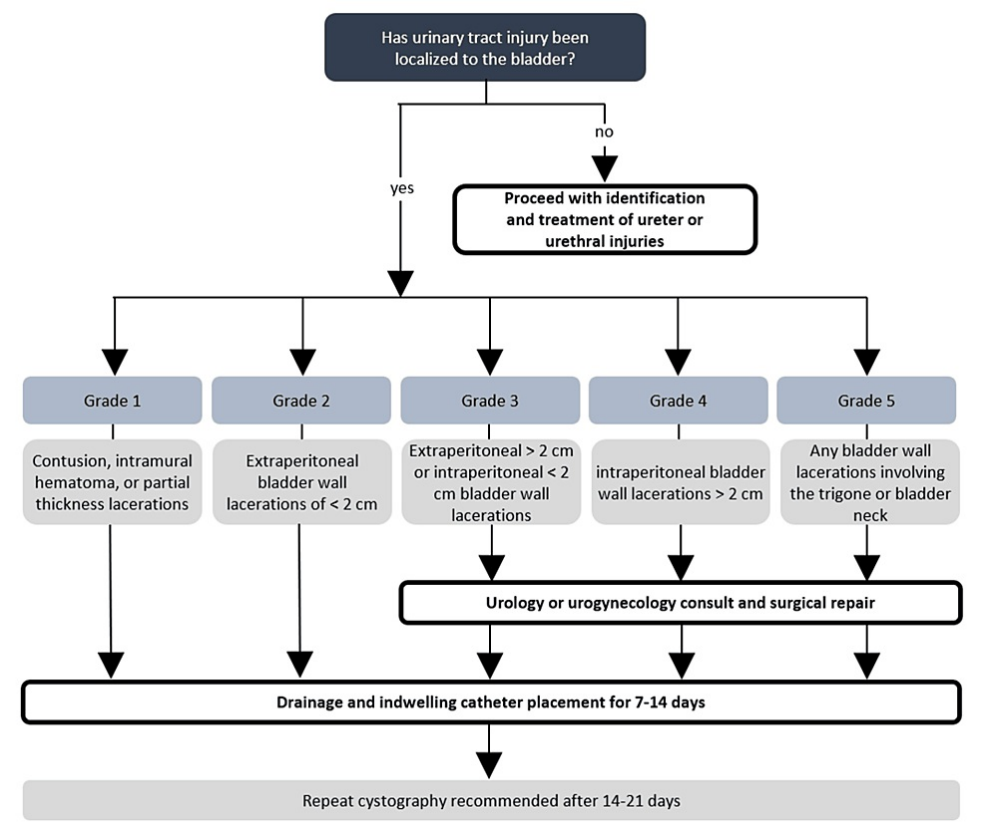
Proposed algorithm for selecting management approach for iatrogenic bladder injuries This is an original figure created by the authors of this report.

Urinary tract injury is treated as soon as diagnosis is confirmed, with treatment approach varying based on the location of injury (ureter, bladder, or urethra) along the urinary tract. Bladder injuries are categorized into five grades: Grade 1 involves contusion, intramural hematoma, or partial thickness lacerations; grade 2 involves extraperitoneal bladder wall lacerations of < 2 cm; grade 3 involves extraperitoneal > 2 cm or intraperitoneal < 2 cm bladder wall lacerations; grade 4 involves intraperitoneal bladder wall lacerations > 2 cm; and grade 5 involves intra- or extra-peritoneal bladder wall lacerations involving the trigone or bladder neck [17]. Grade 1 and grade 2 injuries are managed non-surgically, with drainage and indwelling catheter placement for 7-14 days [17]. Injuries of grade 3 or higher require additional surgical management using polyglactin, poliglecaprone, or plain catgut sutures [17]. An algorithm for the management of various grades of bladder injury is outlined in Figure 3. In the presented case, polyglactin (Vicryl) was used to repair the two grade 3 intraperitoneal defects that were found. Smaller defects < 2 cm, such as those in the presented case, can be repaired with a single-layer closure, while larger ones are preferentially closed with two layers [17]. Of note, in an experimental study comparing saturation time, integrity, and quality of bladder repairs in pig bladders using polyglactin 910 and barbed polyglyconate, closing the bladder with running knotless barbed suture provided faster and more effective watertight closure than polyglactin 910 [18]. Barbed suture should be considered over traditional monofilament materials when possible. For injuries to the bladder trigone, consultation with experienced urological or urogynecological surgeons is highly recommended. In all cases of iatrogenic urinary tract injury, repeat cystography is recommended 14-21 days after surgical and non-surgical management [19]. As seen in the presented patient’s case, a follow-up cystogram was performed after two weeks to confirm repair integrity (Figure 2).

## Conclusions

The presented case illustrates the considerations encountered in the management of a 47-year-old woman with iatrogenic bladder injury that occurred during laparoscopically assisted vaginal hysterectomy for failed medical management of menorrhagia. The intraoperative identification of adhesions and scar tissue from previous surgeries, along with the patient’s high BMI, and the choice of surgical approach were likely contributing factors to the increased complexity of the procedure, leading to the inadvertent creation of a small tear in the bladder. The iatrogenic bladder injury, a grade 3 complication, brings to attention the importance of careful examination and awareness of anatomical variations in overweight patients with a history of extensive abdominal surgeries. The report also highlights the need for a high index of suspicion in diagnosing urinary tract injuries, and understanding the diagnostic approach and subsequent management guidelines when a defect is confirmed. Following up with a cystogram after treatment also highlights the importance of post-operative assessments to ensure integrity of the surgical repair. This case report not only sheds light on one of the most common complications associated with hysterectomy but also emphasizes the importance of thorough anatomical knowledge, comprehensive intra- and post-operative management, and multidisciplinary collaboration.
